# On the road with Henry Lynch

**DOI:** 10.1186/s13053-020-0134-4

**Published:** 2020-01-27

**Authors:** Steven A. Narod

**Affiliations:** 1 Women’s College Research Institute, Women’s College Hospital, 76 Grenville Street, Toronto, ON M5S 1B1 Canada; 2 Dalla Lana School of Public Health, University of Toronto, Toronto, Canada

## On the road with Henry Lynch

One morning in 2003 we piled into Henry Lynch’s sedan and headed north of Omaha to drive 271 miles to Viborg, South Dakota, population 832. There weren’t a lot of cars heading to the Dakotas that Friday and the trip was short and the road was even and we drove straight through except for a bathroom break at Sioux Falls. Henry was driving, Jane Lynch was in the front next to Henry and Carrie Snyder, me and Kelly Metcalfe were settled in the back. Henry’s attention was evenly divided between recalling past family encounters, watching the road and listening to Jane who questioned the accuracy of his reckonings and attended to various administrative matters like calculating the economies of sharing motel rooms and whether or not we could claim for the cost of drinks on the NIH budget (we couldn’t). Jane organized all things – she handled the reservations, venues and the paperwork in general – and a few minutes after we had arrived in town she was passing out our room keys at the Viborg Motel.

We were there to conduct a Family Information Session for a hereditary breast cancer family which we had enlisted in our research program. This was Family 310, one of the founding families in the Creighton University Family Registry. The BRCA1 and BRCA2 genes had been discovered in 1991 and 1994 and we were now trying to enroll more families and to assemble as many potential mutation carriers as possible for our clinical studies and for our epidemiology database. It was a new world.

Next morning we entered our headquarters in the gym of the local community center to prepare for the day. We set up folding chairs and stacked info sheets on a nearby table. The morning session would start with a couple of lectures by Henry and in the afternoon we would counsel family members one-on-one and ask whether or not they wanted to be tested. We had recently found a BRCA1 mutation in a woman in this family and she had organized the Viborg reunion to bring together her family members for testing. The family arrived and each hugged another and the session started with Henry explaining what hereditary breast and ovarian cancer was all about and giving a description of the genetic testing process. Henry in his monogrammed white coat appeared larger than life. This was a big family gathering - seventy or so siblings, aunts uncles, cousins and spouses who listened carefully to Henry’s words - and a couple of stray kids and a grandparent or two who were not quite so attentive. Henry leaned forward towards the audience and his crouch brought him closer to human size. He talked with his shoulder-crushing hands balled into fists to emphasize a point.

We had been doing Family Information Sessions like this for a few years now - each gathering was a road show where we got as many family members together for talking and testing. In the nineties, genetic testing was only done in a few research laboratories and there was a lot of fanfare. Now that testing is mainstream the process is private and much more subdued; there are many more genetic counsellors available and testing can be done secretly for those who wish, by sending a spit sample directly to the laboratory. Pretest one-on-one face-to-face counselling which was once mandatory has slowly morphed into group counselling, then telephone counselling, then instructive on-line videos and finally, for some, with no pre-test counseling at all. Nowadays, genetic counselling is suggested but not mandatory and the majority of women who seek testing privately through a commercial laboratory opt out of pretest counselling even when it is free.

Our Creighton team was the first to give test results to women going as far back as 1992, first with linkage analysis and later by gene sequencing. The pedigree of our largest family, Family 1816, occupies eight pages of finely typed print and genealogic symbols - circles for women and squares for men. This is the largest hereditary breast ovarian cancer family studied to date and the family was instrumental in the mapping of BRCA1 in 1991. When the pedigree was first drawn it spanned five generations and recently we added a sixth. We have blood samples from 151 individuals and have given a test result to 116. There are 29 cases of breast cancer and 14 cases of ovarian cancer so far recorded. We hope there will be no more.

Surprisingly in November 28, 1989, on the day I met Henry Lynch, the genetic link between breast and ovarian cancer was still a matter of speculation. We met in Lyon, France at the international Agency for Research on Cancer (IARC) for a Workshop on linkage studies of hereditary breast cancer [1]. The meeting was organized by my boss, Gilbert Lenoir, who was head of the Unit of Viral and Hereditary Factors in Cancer (neither was considered of sufficient importance to merit its own Unit at the time). All of us experts managed to squeeze around one large round table in Sasakawa Hall. Bruce Ponder entered looking professorial with longish hair and a graduate student named Doug Easton in tow. He was not yet Sir Bruce but we knew it was only a matter of time. Peter Devilee represented the Group from Leiden and Tim Bishop hailed from Leeds. Judy Garber, soft-spoken and petite, was the envoy from the NIH laboratory of Fred Li. Pal Moller flew in from Oslo to stir the pot. Henry was already a legend; he had been in the family cancer business for 20 years by now, but for the rest of us this was new ground. He gave us an introduction to the face of the Hereditary Breast Ovarian Cancer Syndrome, showing slides of multiple pedigrees on the state-of-the-art IARC projector. The atmosphere was collegial, camaraderie was high and offers of sharing data were bandied about. I don’t remember much of what we said that day, but the prix fixé menu that night at the La Tassée restaurant featured a choice of boudin, rabbit or poulet de Bresse.

Henry provided all the families in the Creighton Registry to Gilbert and me and thereby officially kick-started our search for BRCA1. DNA samples were shipped from Omaha to our lab. We began the search in earnest with five breast-ovarian families. We used RFLP markers and Southern blotting and a LOD score mapping approach. We would map first and then clone. In October 1990, at the meeting of the ASHG in Cincinnati, Mary-Claire King announced a positive linkage of familial breast cancer to a marker on chromosome 17q. I was in Cincinnati the night the announcement was made, just down the road from the convention center at Game 2 of the 1990 World Series between the Cincinnati Reds and the Oakland A’s, which the Reds eventually swept.

Back in France, we started testing this marker on the five families from Creighton we had assembled. The largest Lod score was 2.72 for family 1816, confirming the association. Importantly, the testing showed that the gene in question caused ovarian cancer as well. Our paper was published in *The* ***Lancet*** in early 1991 [2].

As they often do, times have changed. With current sequencing technologies, given the same set of DNA samples, I think we could probably find BRCA1 in 2 weeks if we started now. Instead, we are looking for rare genes that account for fewer than 1% of families. In contrast, BRCA1 and BRCA2 together account for about 70% of all hereditary breast cancer families.

After the BRCA1 and BRCA2 genes were identified we switched our attention to clinical and epidemiology studies using the vast stores of information that were in the Creighton database and from other collaborators around the world. We wanted to find out the risk of cancer and how to prevent it. Also how to treat it. I have 17,000 women enrolled in my cohort study from 51 different hospitals around the world and we keep track of about 10,000 of them. Five hundred are from Henry’s Hereditary Cancer Registry in Omaha.

This is a short story of hereditary breast and ovarian cancer and the view as I saw it from Henry’s shoulders. It is for others to tell the story of hereditary colon cancer, now called Lynch syndrome and the SBLA (Sarcoma, Breast, Leukemia, Adrenocortical) syndrome, now called the LI-Fraumeni syndrome. Genetic testing is now routine for breast, ovarian and colon cancer. There is also familial melanoma, but as far as we know the gene for this was never found.

What I liked best about Henry was that he never told me no. Every request for patient data, a pedigree, to review a paper or to write a letter of reference was met with a fast and enthusiastic thumbs up - as predictable as an Iowa cornfield. Over 30 years we have co-authored 151 papers with a couple more on the way. Henry was effusive and like a doting father he heaped praise on our most meagre contributions in an over-the-top enthusiastic style that was uniquely his own. And at the end of each message he always thanked us for the chance to participate. He was a big guy with a big heart but never played the big shot. There are no knighthoods in Nebraska.

Henry started his adult life as a navy man and then engaged in a short boxing career that left him with a scar over one eyebrow but, lucky for us, with all his faculties intact. Henry Lynch was born in Mississippi in 1929 and died in hospice in Omaha in 2019, 7 years after the passing of his wife Jane, not far from the office that they called home. I applaud you Henry for your generous spirit, for a lifetime of hard work and for your infectious enthusiasm. I thank you for choosing the road less travelled and for taking me along on the ride.
